# Predictors for postoperative dysphagia in liver transplant recipients

**DOI:** 10.3389/frtra.2024.1415141

**Published:** 2024-08-16

**Authors:** Marian Isdahl, Lily Katz, Michaela Johnson, Glen Leverson, David Al-Adra, Susan Thibeault

**Affiliations:** ^1^ Department of Surgery, Division of Otolaryngology – Head and Neck Surgery, University of Wisconsin, Madison, WI, United States; ^2^ Department of Brain and Spine, University of Tennessee Medical Center, Knoxville, TN, United States; ^3^ Department of Surgery, University of Wisconsin Madison, Madison, WI, United States; ^4^ Division of Transplantation, Department of Surgery, University of Wisconsin School of Medicine and Public Health, Madison, WI, United States

**Keywords:** dysphagia, liver, transplant, MELD, liver transplantation

## Abstract

**Introduction:**

Liver transplant recipients are at a heightened risk for oropharyngeal dysphagia; identification of those who are at high risk for postoperative dysphagia could reduce hospital costs and length of stay. We sought to identify predictors of dysphagia, in a large cohort of patients who underwent liver transplantation.

**Methods:**

Electronic medical records were queried for patients undergoing liver transplantation, who underwent instrumental swallowing evaluations. Demographics, functional outcomes, and interventions were collected. Logistic regression analyses were performed to identify predictors of dysphagia.

**Results:**

Seven hundred and ninety-five patients met inclusionary criteria. Multivariate analyses found ethnic group (*p* = .0191), MELD Score (*p* < 0001), cold ischemia time (*p* = .0123), and length of intubation (*p* < .0001) to be predictors of post-operative development of dysphagia. Pre-transplant dialysis (*p* < .0001), dysphagia related to end stage liver disease (*p* < .0001), Karnofsky Performance Status Scale (*p* < .0001), wait time to transplant (*p* = 0.0173), surgery time (*p* = 0.0095), tracheostomy (*p* < 0.0001), and transfusion of intraoperative RBC (*p* < .0001), intraoperative platelets (*p* = 0.0018), intraoperative FFP (*p* = 0.0495), perioperative FFP (*p* = 0.0002), perioperative platelets (*p* = 0.0151) and perioperative RBC (*p* = 0.0002) were variables of significance associated with the development of postoperative dysphagia from univariate analysis.

**Conclusions:**

Our results propose a set of predictors that should be considered when identifying post-operative critically ill patients at risk for dysphagia.

## 1 Introduction

Liver transplant recipients (LTR) often suffer from pulmonary complications and prolonged mechanical ventilation, yet the incidence and predictive risk factors for dysphagia in this population remain unknown (1). Dysphagia, defined as disordered or difficulty swallowing, can result in significant delays to hospital discharge, decline in health and in quality of life (QOL). Disruption of neurological or peripheral control of movements involving muscles of the oral cavity, larynx, pharynx, esophagus, or the respiratory system can result in dysphagia. Deviations in physiologic components of normal oropharyngeal swallowing have been linked to aspiration events and predisposition to aspiration pneumonia (2). Studies have demonstrated links between oropharyngeal dysphagia as complications of cerebrovascular accidents, cardiac surgery, prolonged endotracheal intubation, head and neck cancer, and neurodegenerative disease, among others, however relatively few exist regarding the relationship between organ transplantation and postoperative dysphagia (1). Atkins and colleagues assessed oropharyngeal dysphagia after lung transplantation and found a high rate of postoperative dysphagia and silent aspiration (3). Other research has studied the prevalence of swallowing impairment in adults after cardiac surgery and found that tracheal aspiration was prevalent, covert and directly associated with increased morbidity and mortality (4). The present investigation aims to identify predictors of postoperative dysphagia in a large cohort (*n* = 825) of patients who have received liver transplants over a span of 10 years.

In 2019, Mukdad and colleagues studied the utilization and efficacy of a dysphagia screener in LTR, previously used in the cardiac surgical population, with a secondary outcome of determining predictive factors for dysphagia. While a small sample size (*n* = 50) limited their findings, they concluded that older age (average 59 years) and longer postoperative stay were highly associated with the development of postoperative dysphagia. Preoperative creatinine, ammonia levels and Model for End-Stage Liver Disease (MELD) scores at the time of transplantation were not found to have association with dysphagia. Additionally, incidence of hepatic encephalopathy, esophageal varices, and renal failure did not predict the development of postoperative dysphagia (1). Their findings did not suggest a significant difference between increased incidence of dysphagia and longer intubation and operative times, likely due to small sample size (1).

However, given a patient's immunosuppressed state, frailty, neurological/metabolic changes, and complex nature of the surgical procedure, LTR may be at an increased risk for postoperative dysphagia, and at an even heightened risk for aspiration pneumonia. In alignment with the findings of similar cohort studies, we hypothesized that age, intubation time, and operative time may be predictors of postoperative dysphagia after liver transplantation (1, 5, 6). Identification of patients who are at high risk for postoperative dysphagia following liver transplantation will allow medical providers to efficiently screen and involve speech language pathologists (SLPs) earlier in the postoperative period to evaluate and treat patients with the end goal of reducing hospital costs and length of stay (LOS).

## 2 Methods

This retrospective, observational cohort study reviewed the data of 825 patients who underwent a liver transplant at a large tertiary care hospital, between October 2009 and September 2019. Upon approval from the institutional ethics board, pre-, peri- and postoperative data pertaining to the procedure was obtained from the hospital's liver transplant database. Data related to postoperative dysphagia was manually extracted from the electronic medical record (EMR).

All participants underwent a liver transplant from October 2009—September 2019 and had to be equal to or older than 18 years. Exclusionary criteria included history of head and neck surgery with potential for structural or functional alteration to the head and neck, history of dysphagia not related to pre-transplant condition, history of previous liver transplant, and death within 48 h of operation.

### 2.1 Variables

Potential predictive factors for postoperative dysphagia included age, sex (F, M), body mass index (BMI), race (American Indian/Alaskan Native, Asian, Black/African American, Native Hawaiian or other Pacific Islander, White, Decline to Answer), ethnicity (Hispanic/Latino v not Hispanic/Latino), pre-transplant dialysis (Y/N), Karnofsky Performance Status Scale (KPSS), MELD score (6–40), types of transplant donor (live, donation after brain death (DBD), donation after cardiac death (DCD)), cold ischemia time (CIT) (minutes), wait time to transplant (days), surgery time (hours), history of diabetes mellitus (Y/N), history of GERD (Y/N), history of hypertension (Y/N/unknown), length of intubation (hours), dysphagia related to end-stage liver disease (ESLD) (Y/N), tracheostomy (Y/N), number of previous organ transplants (1–4), reason for transplant (alcoholic cirrhosis, liver malignancy, bile duct, metabolic disease, nonalcoholic steatohepatitis, other, viral cirrhosis (VIR/CIR), intraoperative transfusion of red blood cells (RBC) (units), platelets (units), fresh frozen plasma (FFP) (units), and perioperative transfusion of FFP (units), platelets (units), and RBC (units).

As standard of care, post transplantation, patients who exhibited clinical signs/symptoms of dysphagia (e.g., coughing with liquids, throat clearing during meals, etc.) or who had other medical risk factors/indicators (e.g., altered mental status, compromised respiratory status, chest imaging indicative of aspiration, prolonged intubation, etc.) concerning for possible postoperative dysphagia, as determined by the medical team, were referred to a licensed Speech Language Pathologist (SLP). All patients who received SLP referrals were seen within 24 h for a clinical swallow evaluation (CSE) at the bedside. If clinically indicated per the SLP, the patient received an instrumental swallow study (Video Fluoroscopic Evaluation of Swallowing [VFSS] or Flexible Endoscopic Evaluation of Swallowing [FEES]).

Following full evaluation by an SLP, the patient's dysphagia severity was determined using the Dysphagia Outcome and Severity Scale (DOSS) (7). The DOSS is an easy-to-use, 7-point scale that was developed to systematically rate the functional severity of dysphagia based on objective assessment. The DOSS considers both physiologic characteristics, including oral phase deficits, pharyngeal stasis, and extent of airway invasion, based upon a fluoroscopic evaluation, as well as clinical outcomes including level of independence, nutrition, and diet modifications. A lower DOSS score corresponds to a greater severity of dysphagia. DOSS scores were derived from the CSE or instrumental swallow evaluation if completed within 48 h of the CSE. The DOSS scores were coded by an author and a separate, independent blinded rater analyzed a random sample of 10% with 91% inter-rater reliability achieved.

### 2.2 Statistical analysis

Data analysis was performed using SAS statistical analysis software (version 9.4, SAS Institute Inc., Cary, NC). A binary variable of dysphagia was derived from the DOSS to aid in further analysis. Specifically, a DOSS score of 1–5 was defined as known dysphagia, whereas a DOSS of 6–7 was defined as a variant of normal (7). Association between individual factors and the presence of dysphagia on initial post-transplant evaluation were conducted with Fisher's exact tests for categorical variables and *t*-tests for continuous variables. Multivariable models were developed with factors that were significant in these initial tests, where significance was determined based on whether the *p*-value was less than 0.05. Some factors were omitted from the multivariable model because of multicollinearity, or an association with other independent variables.

## 3 Results

### 3.1 Patient characteristics

Of the initial 825 patients, 795 met inclusionary criteria (Table 1). As deemed appropriate from the medical team, a consult for a swallow evaluation by an SLP was received for 264 patients who received a liver transplant. Of the 264 patients evaluated, all received a clinical swallow exam, and 178 patients (67%) received an instrumental swallow evaluation during inpatient admission. Out of the 264 patients evaluated, 218 were found to have dysphagia post-transplantation (83%) based on DOSS scores. Fifty-eight patients were rated as DOSS 5, 62 patients at DOSS 4, 20 patients as DOSS 3, 12 patients as DOSS 2 and 66 patients as DOSS 1. The average age of patients with dysphagia post-transplantation was 55.3 (±11.1) years with an average BMI of 29.4 (±7.0). There were 83 females and 135 males. The average MELD score of patients with dysphagia post-transplantation was 30 (±10.5) as compared to patients without dysphagia, MELD 22.4 (±10.6). The average wait time for patients with dysphagia post-transplantation on the transplant list was 174.1 days (±358.1). Average length of surgery for patients with dysphagia was approximately 7.8 h (±2.5). Patients with dysphagia were intubated for surgery for an average period of 81.6 h (±107.7). The average length of time between transplant and initial swallow consult was 5 days.

**Table 1:** Patient demographics.

	Total population *n* = 795	Post-transplant dysphagia *n* = 218	No Post-transplant dysphagia *n* = 577
**Sex**			
Male	529 (66.5%)	135 (25.5%)	394 (74.4%)
Female	266 (33.5%)	83 (31.2%)	183 (68.8%)
Age	55 (±11)	55.3 (±11.1)	54.9 (±11.0)
BMI	29.8 (±7.1)	29.4 (±7.0)	30.0 (±7.3)
**Race**			
American Indian/Alaskan Native	14 (1.8%)	1 (7.1%)	13 (92.8%)
Asian	12 (1.5%)	4 (33.3%)	8 (66.6%)
Black/African American	27 (3.4%)	10 (37.0%)	17 (62.9%)
Native Hawaiian/Pacific Islander	1 (0.1%)	0 (0%)	1 (100%)
White	738 (92.8%)	202 (27.3%)	536 (72.6)
Decline to Answer	3 (0.4%)	1 (33.3%)	2 (66.6%)
**Ethnicity**			
Hispanic/Latino	36 (4.5%)	3 (8.3%)	33 (91.6%)
Not Hispanic/Latino	756 (95.5%)	214 (28.3%)	542 (71.6%)
**Pre-transplant dialysis**			
Yes	175 (22.4%)	94 (53.7%)	81 (46.2%)
No	606 (77.6%)	117 (19.3%)	489 (80.6%)
**KPSS**			
Moribund, fatal processes progressing rapidly – 10%	77 (9.7%)	53 (68.8%)	24 (31.1%)
Very sick, hospitalization necessary; active treatment necessary – 20%	127 (16%)	60 (47.2%)	67 (52.7%)
Severely disabled; hospitalization is indicated, death not imminent - 30%	160 (20%)	53 (33.1%)	107 (66.8%)
Disabled; requires special care and Assistance – 40%	241 (30.3%)	33 (13.6%)	208 (86.3%)
Requires considerable assistance and frequent medical care – 50%	62 (7.8%)	8 (12.9%)	54 (87.1%)
Requires occasional assistance but is able to care for needs – 60%	55 (6.9%)	7 (12.7%)	48 (87.2%)
Cares for self; unable to carry on normal activity or active work – 70%	35 (4.4%)	3 (8.5%)	32 (91.4%)
Normal activity with effort; some symptoms of disease – 80%	32 (4%)	1 (3.1%)	31 (96.8%)
Able to carry on normal activity; minor symptoms of disease – 90%	6 (0.8%)	0 (0%)	6 (100%)
MELD	24.5 (±11)	30.0 (±10.5)	22.4 (±10.6)
**Type of transplant donor**			
Live	23 (2.9%)	5 (21.7%)	18 (78.2%)
DBD	669 (84.2%)	193 (28.8%)	476 (71.1%)
DCD	103 (13%)	20 (19.4%)	83 (80.5%)
Cold ischemia time (minutes)	7 (±2.3)	7.5 (±2.5)	6.9 (±2.3)
Wait time to transplant (days)	230.3 (±480.4)	174.1 (±358.1)	251.6 (±517.9)
Surgery time (hours)	7.5 (±2.2)	7.8 (±2.5)	7.4 (±2)
**History of Diabetes Mellitus**			
Yes	223 (28%)	52 (23.3%)	171 (76.6%)
No	572 (71.9%)	166 (29%)	406 (70.9%)
**History of GERD**			
Yes	137 (17.2%)	43 (31.3%)	94 (68.6%)
No	658 (82.8%)	175 (26.6%)	483 (73.4%)
**History of HTN**			
Yes	411 (52.2%)	107 (26%)	304 (73.9%)
No	371 (47.1%)	109 (29.3%)	262 (70.6%)
Unknown	5 (1%)	0 (0%)	5 (100%)
Length of intubation (hours)	42.9 (±72.2)	81.6 (±107.7)	28.4 (±45.4)
**Dysphagia related to ESLD**			
Yes	51 (6.4%)	37 (72.5%)	14 (27.45%)
No	743 (93.5%)	180 (24.2%)	563 (75.8%)
**Tracheostomy**			
Yes	26 (3.3%)	22 (84.6%)	4 (15.3%)
No	769 (96.7%)	196 (25.4%)	573 (74.5%)
**# of previous organ transplants**			
0	740 (93.1%)	197 (26.6%)	543 (73.3%)
1	46 (5.8%)	17 (36.9%)	29 (63%)
2	8 (1%)	3 (37.5%)	5 (62.5%)
3	1 (0.1%)	1 (100%)	0 (0%)
Intra-op RBC (Units)	9.1 (±12.4)	13.3 (±16.3)	7.5 (±10.1)
Intra-op Platelets (Units)	8.5 (±35.9)	15.1 (±65.7)	6 (±11.5)
Intra-op FFP (Units)	6.5 (±14.9)	9.8 (±15.7)	5.3 (±14.3)
Peri-op FFP (Units)	1.0 (±2.5)	1.5 (±3.3)	0.8 (±2.1)
Peri-op Platelets (Units)	2.9 (±10.1)	4.8 (±16)	2.1 (±6.47)
Peri-op RBC (Units)	2.1 (±3.2)	2.8 (±4.1)	1.8 (±2.8)
**Reason for transplant**			
Alcoholic cirrhosis	321 (40.4%)	94 (29.2%)	227 (70.7%)
Liver malignancy	107 (13.5%)	15 (14%)	92 (85.9%)
Bile duct	115 (14.5%)	33 (28.7%)	82 (71.3%)
Metabolic disease	20 (2.5%)	7 (35%)	13 (65%)
Non-alcoholic steatohepatitis	110 (13.8%)	33 (30%)	77 (70%)
Other	43 (5.4%)	15 (34.8%)	28 (65.1%)
VIR/CIR	79 (9.9%)	21 (26.5%)	58 (73.4%)

BMI, body mass index; KPSS, Karnofsky performance status scale; MELD, model for end stage liver disease; DBD, donation after brain death; DCD, donation after cardiac death; GERD, gastroesophageal reflux disease; HTN, hypertension; ESLD, end stage liver disease; RBC, red blood cells; FFP, fresh frozen plasma; VIR/CIR, viral cirrhosis.

### 3.2 Predictors of dysphagia development

An initial univariate analysis found 15 variables statistically significant out of the original 26 potential predictive factors as outlined in Table 2. These include Hispanic/Latino (*p* = 0.0158), pre-transplant dialysis (*p* < 0.0001), KPSS (*p* < 0.0001), MELD (*p* < 0.0001), cold ischemia time (*p* = 0.0022), surgery time (*p* = 0.0095), length of intubation (*p* < 0.0001), dysphagia related to ESLD (*p* < 0.0001), tracheostomy (*p* < 0.0001), intraoperative transfusion of RBCs (<0.0001), platelets (*p* = 0.0018), FFP (*p* = 0.0495), and perioperative transfusion of FFP (*p* = 0.0002), platelets (*p* = 0.0151), and RBCs (*p* = 0.0002). Table 2 includes odds ratios and confidence intervals for all variables.

**Table 2:** Univariate regression analysis for dysphagia.

Variable	*p*-value	OR	CI
Female	0.0906	1.32	0.96–1.83
Age	0.6450	1.00	0.99–1.02
BMI	0.6969	0.99	0.97–1.02
Caucasian	0.9087	1.04	0.57–1.89
Hispanic/Latino	0.0158*	0.23	0.07–0.76
Pre-transplant dialysis	<0.0001*	4.85	3.39–6.95
KPSS^a^	< 0.0001*		
10%		68.46	8.82–531.18
20%		27.76	3.68–209.60
30%		15.53	2.04–115.56
40%		4.92	0.65–37.26
50%		4.59	0.55–38.46
60%		4.52	0.53–38.56
70%		2.91	0.29–29.47
MELD	<0.0001*	1.07	1.05–1.09
Type of transplant donor^b^	0.1162		
DBD		1.44	0.53–3.99
DCD		0.87	0.288–2.62
Cold ischemia time	0.0022*	1.12	1.04–1.19
Wait time to transplant	0.1304	1.00	0.99–1.00
Surgery time	0.0095*	1.10	1.02–1.18
History of diabetes mellitus	0.1061	0.744	0.52–1.07
History of GERD	0.2534	1.26	0.85–1.88
History of HTN	0.5789	0.846	0.62–1.16
Length of Intubation	<0.0001*	1.02	1.01–1.02
Dysphagia related to ESLD	<0.0001*	8.26	4.37–15.63
Tracheostomy	<0.0001*	16.078	5.47–47.23
# of previous organ transplants^c^	0.4391		
1		1.62	0.87–3.01
2		1.65	0.39–6.98
Intraoperative RBC	<0.0001*	1.04	1.02–1.05
Intraoperative platelets	0.0018*	1.02	1.01–1.03
Intraoperative FFP	< 0.0001*	1.04	1.02–1.06
Perioperative FFP	0.0002*	1.13	1.06–1.20
Perioperative platelets	0.0151*	1.03	1.00–1.05
Perioperative RBC	0.0002*	1.10	1.05–1.16
Reason for transplant^d^	0.0662		
Alcoholic cirrhosis		1.14	0.66–1.99
Liver malignancy		0.45	0.22–0.95
Bile duct		1.11	0.59–2.11
Metabolic disease		1.49	0.52–4.23
Non alcoholic steatohepatitis		1.18	0.62–2.26
Other		1.48	0.66–3.30

BMI, body mass index; KPSS, Karnofsky performance status scale; MELD, model for end stage liver disease; DBD, donation after brain death; DCD, donation after cardiac death; GERD, gastroesophageal reflux disease; HTN, hypertension; ESLD, end stage liver disease; RBC, red blood cells; FFP, fresh frozen plasma; VIR/CIR, viral cirrhosis.

^a^ OR referent is 80%.

^b^ OR referent is Live.

^c^ OR referent is 1.

^d^ OR referent is VIRCIR.

* *p* < 0.05.

From the significant univariate variables, a multivariable logistic regression model was determined (Table 3). After the omission of variables due to multicollinearity, the following variables were included: Hispanic/Latino, MELD, wait time to transplant, cold ischemia time, intraoperative RBC, surgery time, length of intubation, and perioperative RBC transfusion. Hispanic/Latino (*p* = 0.0191, OR =.18), MELD (*p* < .0001, OR 1.06), cold ischemia time (*p* = 0.0123, OR 1.10), and length of intubation (*p* < .0001, OR = 1.01) were found to be significant. Wait time to transplant (*p* = 0.7674), surgery time (*p* = 0.7666), intraoperative RBC (*p* = 0.2245), and perioperative RBC (*p* = 0.4515) were not found to be significant. C-index for the multivariate model was.806, indicating a strong model to predict postoperative dysphagia.

**Table 3:** Multivariate analysis for dysphagia.

Effect	Significance	OR	CI
Hispanic/Latino	0.0191*	0.18	0.04–0.75
MELD	<.0001*	1.06	1.04–1.08
Wait time to transplant	0.7674	1.00	1.00–1.00
Cold ischemia time	0.0123*	1.10	1.02–1.20
Intraoperative RBC	0.2245	1.01	0.99–1.03
Surgery time	0.7666	0.99	0.90–1.08
Length of intubation	<.0001*	1.01	1.01–1.02
Perioperative RBC	0.4515	1.02	0.97–1.08

MELD, model for end stage liver disease; RBC, red blood cells.

* *p* < 0.05.

## 4 Discussion

The aim of this investigation was to identify predictors of oropharyngeal dysphagia in a large cohort of patients following a liver transplant. Overall, our significant findings indicate the factors that relate to a patient's health and fragility prior to transplant as effective predictors to evaluate a patient's risk for developing oropharyngeal dysphagia. Our multivariate findings suggest the more advanced a patient's illness is prior to organ transplant (e.g., higher MELD score), or the longer total time intubated during and after the surgery, the higher the risk for developing post-transplant dysphagia. These factors should be taken into consideration when determining the appropriateness for a referral to SLPs specializing in evaluation and treatment of oropharyngeal dysphagia.

MELD score is used to determine need for liver transplant and indicates likelihood of mortality in individuals with end-stage liver disease (8). In this investigation, for every one-point increase in MELD score, the odds of developing dysphagia post-transplantation increased 1.06 times. Dysphagia in patients with a higher pre-transplant MELD is more than likely secondary to the overall severity of liver disease. A higher MELD score indicates a more advanced disease with a corresponding increase in medical complications, such as encephalopathy (9, 10). Patients who are more decompensated prior to significant surgical intervention are at a higher risk of having complications and a slower recovery, including the ability to safely swallow food and liquid. MELD as a predictor is highly relevant; it is widely used and recorded in the EMR, which makes it easy for all providers to use to identify the patients who should be evaluated for dysphagia by an SLP.

CIT is a known donor risk factor, with research showing high CIT increases the risk of prolonged length of hospital stay (11). In our cohort, a significant association was found between CIT and risk of developing dysphagia post-transplantation. The average CIT with resultant dysphagia was 7.5 h, compared to the average CIT without resultant dysphagia, which was 6.8 h. For every one-hour increase in CIT, the odds of developing dysphagia post-transplantation increased 1.10 times. This finding highlights the importance of assessing donor risk factors, in conjunction with recipient risk factors, when deciding if a dysphagia evaluation prior to diet initiation would benefit long-term outcomes post transplantation.

Prolonged intubation (defined as intubation >48 h), and mechanical ventilation have been highly associated with various complications ranging from nasal necrosis, hyperthermia, gastric distention, tracheal erosion, pneumothorax, ventilator acquired pneumonia, among others (12). Current research suggests patients are additionally at high risk for developing oropharyngeal dysphagia following prolonged endotracheal intubation (4, 13–15). In our cohort, for every 24 h of additional intubation, the odds of developing oropharyngeal dysphagia increased 1.01 times. Similarly, studies have shown that prolonged surgery time is associated with increased odds of overall functional complications, along with increased length of stay and return to the operating room (16).

Surprisingly, those patients who were Hispanic/Latino were found to have less likelihood of developing dysphagia, with an odds ratio of <0.2. Those who were not in the Hispanic/Latino group were found to be at a higher risk of developing dysphagia post-transplantation. We suspect that our overall small number of Hispanic/Latino patients (36) compared to Nonhispanic/Latino (756) patients in our cohort is responsible for this finding. Further investigation with a larger number of Hispanic/Latino patients is necessary.

The univariate predictors warrant discussion. The Karnofsky Performance Status Scale (KPSS) is an assessment tool that has been widely used and evaluated by medical professionals since its development in 1948, aimed to measure a patient's functional impairment (17). In our cohort, there was a significant relationship between low KPSS scores and an increased likelihood of dysphagia; patients with KPSS scores in the moribund and/or very sick range were 15–68 times more likely to have dysphagia post transplantation, suggesting that patients with low KPSS scores may benefit from an early swallow evaluation.

The use of blood products, specifically RBC, FFP, and platelets, are used to compensate for blood loss during surgery as well as patient's clotting factors. Historically, the more severe the disease progresses and the poorer hemostatic capabilities of the LTR, the higher the need for increased blood products during surgery (18). Additionally, the use of multiple blood products during surgery can result in tissue edema in the body, including the oropharynx, which can contribute to postoperative dysphagia (19, 20). In our cohort, an increased use of blood products in the intraoperative and perioperative period was found to be a statistically significant predictor of dysphagia following transplant.

Patients with advanced chronic kidney disease (CKD), end stage renal disease (ESRD) and acute kidney injury (AKI) who require intensive medical care such as dialysis have been found to have increased muscle weakness and lack of overall functional endurance (21). Kidney disease often results in progressive deconditioning due to multiple factors (21). In our cohort, the odds of developing dysphagia post-transplantation in those patients with a pre-transplant dialysis increased 4.85 times. This could indicate an overall functional deconditioning of muscles, which then has the potential to impact approximately 25 pairs of muscles in the aerodigestive tract (22).

There were some patients in our cohort who were not appropriate for per os (PO) intake in the immediate time prior to receiving a liver transplant, (i.e., reduced alertness or encephalopathy etc.), as determined by the primary care team. These patients relied on enteral feeding (e.g., post-pyloric feeding tube) to meet nutritional needs leading up to their transplant. Inability to have PO intake prior to transplant (i.e., dysphagia related to ESLD) was found to be a statistically significant predictor of dysphagia post-transplantation. Reduced alertness or encephalopathy, which prevents a patient from safely consuming food and liquid by mouth, is an overall indicator of severity of disease progression (23). It is well documented in current literature that prolonged disuse of swallowing musculature, such as periods of nil per os (NPO) status, lead to muscle atrophy (24). This atrophy can result in impairments in swallow safety and efficiency. Swallowing muscle disuse atrophy is likely a contributing factor in the development of dysphagia with patients who had limited-to-no PO intake prior to surgery. In our cohort, the odds of developing dysphagia post-transplantation in those patients with a history of dysphagia related to end stage liver disease was 8.26 times that of patients without a history of dysphagia related to end stage liver disease.

It is well documented that medically complex patients with the presence of a tracheostomy tube are at an increased risk for developing dysphagia not because of the physical tracheostomy itself, but rather as it relates to the underlying medical condition leading to the need for tracheostomy (e.g., hypoxic respiratory failure, disuse atrophy, laryngeal malignancy etc.) (25–27). Presence of tracheostomy tubes can additionally lead to reduction in subglottic pressure if uncapped (25, 26). Many patients with tracheostomy tubes are at increased risk for aspiration and related complications and benefit significantly from instrumental swallowing evaluations (26). In our cohort, the odds of developing dysphagia post-transplantation in those patients with the presence of a tracheostomy tube was 16.078 times that of patients without the presence of a tracheostomy tube.

There are many factors that influence the amount of time patients wait on the liver transplant list, such as medical urgency, age, and location. Research has shown that survival is higher in patients who are on the waitlist for less time (28). In our cohort, length of wait time until transplantation was found to be a statistically significant predictor of dysphagia. One of the most influential factors determining length of time spent on the transplant waiting list is the medical urgency of the transplant, measured by MELD (29). It was found that less time spent on the waitlist was associated with an increased risk for dysphagia post transplantation. This association is likely secondary to the overall severity of the disease process, like the association discussed above with MELD scores. Patients with a more advanced disease process are likely to be placed higher on the waiting list, thus shortening the wait time, while still being at risk for complications post transplantation including oropharyngeal dysphagia. These results indicate that patients who are high on the liver transplant list, (i.e., those who do not wait long for a liver transplant) could benefit from early dysphagia screening and intervention from an SLP in the early post-operative period.

This study has several limitations which warrant further discussion. Given the retrospective structure of this investigation, not all patients included were referred for a dysphagia evaluation and were thus assumed to have functional oropharyngeal swallowing. As practice patterns differ amongst medical personnel, screenings, and referrals for SLP consults varied. Additionally, of those seen by an SLP, only 67% received an instrumental swallow evaluation during their hospitalization. As a result of these two factors, silent aspiration and the presence or absence of dysphagia cannot be ruled out for patients who did not receive a referral to SLP services and an instrumental swallow evaluation. Since instrumental swallow evaluations were completed up to 48 h after the initial CSE, there is the possibility for improvement between the CSE and instrumental evaluation. Given the acute nature of the patient's medical status, some patients also demonstrated fluctuating dysphagia severity over the course of their hospitalization due to a variety of factors (e.g., acute onset of stroke, respiratory changes, onset of delirium, etc.). Because of this, their initial DOSS score may have reflected milder or a lack of dysphagia depending on the patient's medical status at the time of SLP evaluation.

## 5 Conclusion

In conclusion, we have identified multiple factors that predict the development of dysphagia post-liver transplantation. Through earlier referral of at-risk patients to SLP for assessment, we aim to decrease complications of dysphagia in this vulnerable population.

## Data Availability

The original contributions presented in the study are included in the article/Supplementary Material, further inquiries can be directed to the corresponding author.
